# Amikacin: Uses, Resistance, and Prospects for Inhibition

**DOI:** 10.3390/molecules22122267

**Published:** 2017-12-19

**Authors:** Maria S. Ramirez, Marcelo E. Tolmasky

**Affiliations:** Center for Applied Biotechnology Studies, Department of Biological Science, California State University Fullerton, Fullerton, CA 92831, USA; msramirez@fullerton.edu

**Keywords:** aminoglycosides, antibiotic resistance, amikacin, aminoglycoside modifying enzymes, antisense

## Abstract

Aminoglycosides are a group of antibiotics used since the 1940s to primarily treat a broad spectrum of bacterial infections. The primary resistance mechanism against these antibiotics is enzymatic modification by aminoglycoside-modifying enzymes that are divided into acetyl-transferases, phosphotransferases, and nucleotidyltransferases. To overcome this problem, new semisynthetic aminoglycosides were developed in the 70s. The most widely used semisynthetic aminoglycoside is amikacin, which is refractory to most aminoglycoside modifying enzymes. Amikacin was synthesized by acylation with the l-(−)-γ-amino-α-hydroxybutyryl side chain at the C-1 amino group of the deoxystreptamine moiety of kanamycin A. The main amikacin resistance mechanism found in the clinics is acetylation by the aminoglycoside 6′-*N*-acetyltransferase type Ib [AAC(6′)-Ib], an enzyme coded for by a gene found in integrons, transposons, plasmids, and chromosomes of Gram-negative bacteria. Numerous efforts are focused on finding strategies to neutralize the action of AAC(6′)-Ib and extend the useful life of amikacin. Small molecules as well as complexes ionophore-Zn^+2^ or Cu^+2^ were found to inhibit the acetylation reaction and induced phenotypic conversion to susceptibility in bacteria harboring the *aac(6′)-Ib* gene. A new semisynthetic aminoglycoside, plazomicin, is in advance stage of development and will contribute to renewed interest in this kind of antibiotics.

## 1. A Brief History of Aminoglycoside Antibiotics

**Figure 1:**
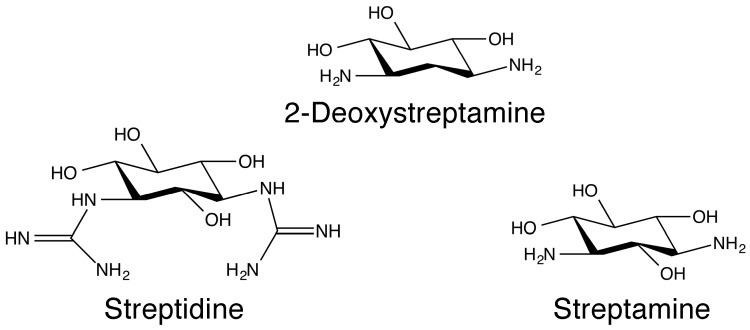
Chemical structures of aminocyclitols.

**Figure 2:**
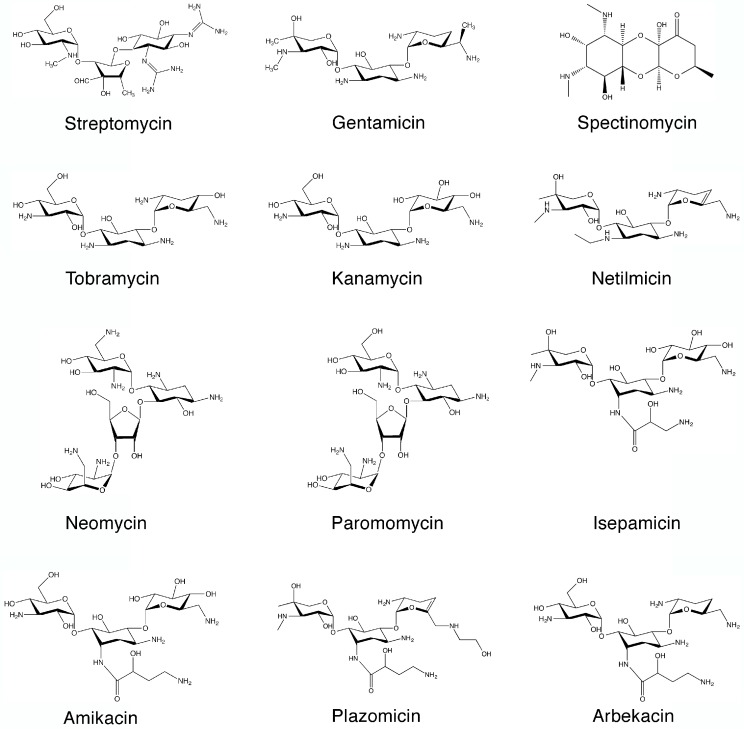
Chemical structures of representative aminoglycosides.

Aminoglycosides are a group of antibiotics primarily used to treat a wide spectrum of bacterial infections [1,2,3,4]. However, modern medicine has found other uses for these agents that include treatments for various genetic disorders and Meniere’s disease [5,6,7,8]. In addition, aminoglycosides are being researched as inhibitors of reproduction of the HIV [3,9]. The general structure of aminoglycosides consists of an aminocyclitol nucleus (streptamine, 2-deoxystreptamine, or streptidine) (Figure 1) linked to amino sugars. In addition, there are few exceptions where the antibiotic is considered an aminoglycoside despite not strictly conforming to this rule such as spectinomycin (Figure 2), which is an aminocyclitol not bound to amino sugars [10]. The first aminoglycoside, streptomycin (Figure 2), was discovered in the early days of the antibiotic era (1944) and it is still in use [11,12]. This discovery was followed by those of neomycin (1949) [13,14], and kanamycin (1957) [15] and gentamicin [16,17] (Figure 2). Following these findings, other natural aminoglycosides such as tobramycin [18] (Figure 2), with robust activity, were discovered. Furthermore, some of them were found to be useful as antifungal and antiparasitic agents [19,20,21,22]. For example, paromomycin (Figure 2), is used in the treatment of cryptosporidiosis, leishmaniasis, and other infections caused by protozoa and cestodes [20]. All natural aminoglycosides in use to date are produced by soil bacteria belonging to the genera *Streptomyces* or *Micromonospora*, and the origin of each one of them is identified by the suffix, “-mycin” and “-micin”, respectively [23,24].

**Scheme 1:**
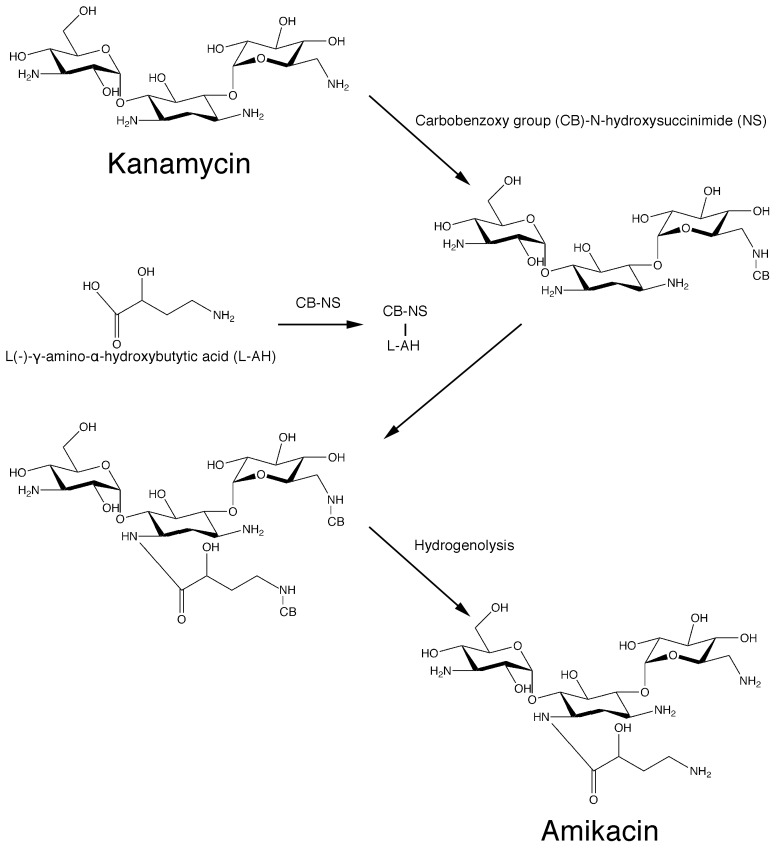
Original chemical reactions sequence to obtain amikacin by modification of kanamycin A.

Recent advances have permitted us to understand the biosynthetic pathways of natural aminoglycosides [25]. Unfortunately, as it is the case with all antibiotics, bacteria developed several mechanisms of resistance that threaten the use of these drugs. Aminoglycoside modifying enzymes, which catalyze inactivation by acetylation (aminoglycoside acetyltransferases, AAC), phosphorylation (aminoglycoside phosphotransferases, APH), or adenylylation (aminoglycoside nucleotidyl-transferases, ANT) of the molecule, are the leading cause of the rapid increase and dissemination of resistance [26,27,28,29,30]. The first enzyme of this kind was identified in 1967 in an *Escherichia coli* strain that possessed an enzyme that could inactivate kanamycin by transferring an acetyl group to the 6′-*N* position of the antibiotic molecule. The resulting compound, 6′-*N*-acetylkanamycin, does not have antibiotic properties [31]. Aminoglycoside 6′-*N*-acetyltransferases belong to the GNAT (GCN5-related *N*-acetyltransferases) superfamily of enzymes, which includes more than 100,000 members found in prokaryotes, eukaryotes, and archaea [32,33], remain a very significant cause of failure of treatment of numerous severe infections [26,34]. More than hundred aminoglycoside modifying enzymes have been identified to date [26,27]. Among the multiple approaches tried to address the problem of resistance caused by aminoglycoside modifying enzymes, modification of the aminoglycoside molecule was among the most successful [35]. Addition of chemical groups that do not impair the antibiotic activity of the aminoglycoside produced new compounds that are not substrate of most aminoglycoside modifying enzymes [35]. These new molecules derived from natural aminoglycosides are known as semisynthetic. The first aminoglycoside of this kind to be used as an antibacterial was dibekacin, introduced in 1975 and still in use in various countries [36,37]. In the following years, numerous semisynthetic aminoglycosides were synthesized with activity against resistance caused by different aminoglycoside modifying enzymes. Amikacin, one of the most successful semisynthetic aminoglycosides, was synthesized by acylation with the l-(−)-γ-amino-α-hydroxybutyryl side chain at the C-1 amino group of the deoxystreptamine moiety of kanamycin A [38] (Scheme 1).

This antibiotic was introduced in 1977, and it is still used with great success to treat a variety of infections although the rise of aminoglycoside 6′-*N*-acetyltransferases type I are limiting its effectiveness [26,34,39,40,41,42,43]. Other pathways for synthesis of amikacin by modification of kanamycin A were later proposed [44,45]. Netilmicin [46] (Figure 2), isepamicin [47] (Figure 2), and arbekacin [48] (Figure 2), introduced in 1985, 1988, and 1990, respectively, are other semisynthetic aminoglycosides that were successfully used to treat resistant infections. Following these developments, there was a period with relatively few additions to the field of aminoglycosides followed by another period characterized by new approaches that took advantage of the deeper understanding of different aspects of the biology and structure of aminoglycoside modifying enzymes as well as the advances in synthetic chemistry. As a consequence, numerous new generation aminoglycosides, also known as neoglycosides, started to be synthesized [19,49,50,51,52,53,54,55,56,57,58]. Of the several neoglycosides existing in the pipeline, plazomicin (ACHN-490) (Figure 2), which has been granted Breakthrough Therapy designation by the FDA in May 2017, is the one closest to be approved for human use [59,60,61,62]. A New Drug Application for plazomicin was submitted in October 2017 to the U.S. Food and Drug Administration [63]. This antibiotic is active against multidrug resistant *Enterobacteriaceae*, including problematic carbapenem- and polymyxin-resistant isolates as well as *Acinetobacter baumannii* [64,65,66,67]. The activity against *Pseudomonas* is similar to or slightly lower than other aminoglycosides [68]. Plazomicin is also active against methicillin-susceptible and methicillin-resistant *Staphylococcus aureus* [68,69].

Of the numerous aminoglycosides known to date, five (amikacin, gentamicin, neomycin, streptomycin, and tobramycin) are listed in the British National Formulary for clinical use in the United Kingdom [70] and amikacin, gentamicin, neomycin, streptomycin, kanamycin, paromomycin, and tobramycin are approved by the US Food and Drug Administration (FDA) for clinical use in the United States [19].

## 2. Mechanism of Action and Side Effects

Due to their polycationic nature, aminoglycosides first bind to the anionic compounds found in the bacterial surface. In the case of Gram-negative bacteria, these compounds are lipopolysaccharide, phospholipids, and outer membrane proteins and in the case of Gram-positives, they are mainly teichoic acids and phospholipids. These interactions produce an increase in permeability that results in penetration of some aminoglycoside molecules into the periplasmic space. This energy-independent mechanism is known as “self-promoted uptake” [71]. Following, in an energy-dependent process, the “energy-dependent phase I”, a small number of molecules of the antibiotic reach the cytoplasm with the participation of a functional electron transport system [72,73,74]. The aminoglycoside molecules inside the cytoplasm produce the antibiotic effect (see below), which results in mistranslated proteins. As a consequence, aberrant cytoplasmic membrane proteins induce damage to the integrity of the cytoplasmic membrane facilitating the entry of aminoglycoside molecules in abundant quantities. This third stage is known as “energy-dependent phase II” [75,76,77,78,79]. The high number of aminoglycoside molecules within the cell produces high levels of errors in protein synthesis leading to more damage in the cytoplasmic membrane permitting a still higher rate of uptake that ultimately results in death of the cell.

Aminoglycosides exert their action through binding to the 30S bacterial ribosome subunit changing the conformation of the A site to one that resembles that one induced by interaction between cognate tRNA and mRNA. As a consequence, proofreading capabilities of the ribosome are reduced, increasing mistranslation [1,76,80,81,82,83,84,85,86,87]. However, although the effect of binding to the ribosome is similar for all aminoglycosides, not all classes of these antimicrobials bind to identical sites of the 16S rRNA. Other effects that may or may not be secondary to RNA binding and protein mistranslation are inhibition of the 30S ribosomal subunit assembly (neomycin and paromomycin) [88,89], ribozyme-like activity resulting in cleavage of RNA molecules [90,91,92] or interference with essential functions dependent on ribozyme activity such inhibition of ribonuclease P [91,93,94].

Aminoglycosides have also been shown to cause other disruptions to bacterial cells when present at subinhibitory concentrations. Goh et al. showed that aminoglycosides at subinhibitory concentrations could modify transcription rates [95] and Possoz et al. found that amikacin at these low concentrations disrupts formation of the Z ring leading to inhibition of cell division [96].

While aminoglycosides were, and continue to be an essential component in the battery of resources to treat severe bacterial infections, their use is not free of side effects. The main toxicity risks are ototoxicity, nephrotoxicity, and rarely neuromuscular blockade [26,97,98,99,100,101,102]. The ototoxicity effects include permanent bilaterally severe, high-frequency sensorineural hearing loss and temporary vestibular hypofunction. The permanent hearing loss occurs as a result of damage caused to the sensory hair cells in the inner ear, in particular, the basal, high-frequency outer hair cells [103,104,105]. Efforts to limit ototoxic effects of aminoglycosides identified several candidates such as free radical scavengers as well as iron chelators [103], salicylate [106], *N*-acetylcysteine [107], and more recently d-tubocurarine and berbamine as potential otoprotectans [108]. Nephrotoxicity caused by aminoglycosides is usually reversible; its main clinical manifestation is nonoliguric acute kidney injury caused by decreased glomerular filtration [26,97,109,110]. Other manifestations include aminoaciduria, glycosuria, hypomagnesemia, hypocalcemia, and hypokalemia. There have been numerous studies to identify compounds that can prevent aminoglycoside nephrotoxicity. A recent systematic meta-analysis of available data recognized 40 chemicals with nephroprotectant activity [111]. Neuromuscular blockade is a rare aminoglycoside toxic effect [97].

## 3. Amikacin

Due to its property of being refractory to most aminoglycoside modifying enzymes, amikacin has been successfully used to treat otherwise aminoglycoside resistant infections, and it is the most widely used semisynthetic aminoglycoside [42,112,113,114,115,116,117,118]. Its pharmacokinetics is similar to that of the natural gentamicin and tobramycin, 30–60 min after intravenous administration there is a peak in serum concentration [97]. The optimal antibacterial effects occur when the maximum concentration in serum is 8 to 10 times higher than the minimal inhibitory concentration (MIC) [97]. Amikacin alone or in combination with other antibiotics is used to treat a variety of serious infections caused by aerobic Gram-negative bacteria, as well as *mycobacteria and Nocardia* [24,114,119,120,121,122,123,124]. This antibiotic is also essential in the treatment of life-threatening infections in neonates [42,115,125,126,127]. Structural studies showed that while amikacin binds the A site of the 16S RNA similarly when compared to kanamycin A, there are specific interactions between the l-(−)-γ-amino-α-hydroxybutyryl group and the RNA at the GC pairs C_1404_–G_1497_ and G_1405_–G_1496_ [128,129].

Amikacin is mainly administered intravenously, intramuscularly, through nebulization [130,131,132,133,134,135,136,137]. Other routes of administration for specific infections are intrathecal or intraventricular [138,139]. Amikacin is mostly administered as a weight-based dose divided in two to three applications per day or as a once-daily strategy, with this latter strategy being the preferred option [123,140,141,142,143]. Since amikacin exhibits the toxic effects common to aminoglycosides, i.e., ototoxicity and nephrotoxicity, the dose regime to maximize therapeutic outcomes and minimize adverse consequences is of great importance. However, a recent systematic study comparing the information available in the literature was inconclusive concerning optimal dosage regimes [144]. A recent review of the population pharmacokinetic models for amikacin described in critically ill patients contributed information to help optimizing amikacin dosage. In particular, the conclusions point against the “one dose fits all” strategy [114]. Amikacin is used to treat infections in neonates, including preterm neonates [42,125,145,146,147]. Although it has been successful in treating infections caused by multidrug resistant strains [126,148,149] there are still controversies about dosage and pharmacokinetics [126,146]. Unfortunately, the recent rise in resistance to amikacin limits the effectivity of many interventions during outbreaks of infection in neonates [41,42,150].

Amikacin was also researched as a formulation in unilamellar liposomes (MiKasome) [151]. However, in spite of early promising results in treatments of several conditions such as urinary tract infection [152], endocarditis [153], *Klebsiella pneumoniae* and *Mycobacterium* infections [154,155,156], the development of the formulation was discontinued in the year 2000.

Among the aminoglycosides currently available for use in humans, amikacin is the most resistant to the action of aminoglycoside modifying enzymes [27,157,158]. However, after it was introduced in the late 70s, resistant strains started to appear in different geographical regions and in some of them it became dangerously high [23,26,27,159,160]. A plasmid-mediated acetyltransferase, now known as AAC(6′)-Ib or AacA4 [26,27,34,161], was first reported in *P. aeruginosa* that conferred resistance to amikacin besides other aminoglycosides but not gentamicin C_1_ [160,162,163]. Early work also identified a plasmid-borne phosphotransferase and chromosomal mutations that resulted in resistance to amikacin in non-clinical *E. coli* strains [164,165,166,167], and a plasmid-mediated adenylyltransferase present in *K. pneumoniae*, *E. coli*, *Serratia marcescens*, and *Proteus vulgaris* strains that could use amikacin as substrate [168]. Amikacin resistance due to decreased uptake was also reported in *K. pneumoniae* [169]. The first documented outbreak of hospital infection with amikacin-resistant *Enterobacteriaceae* in newborn infants occurred in 1978 in the Louisville General Hospital, and three out of 11 neonates infected died [170]. Different *Mycobacterium* species developed resistance to amikacin through substitutions in the ribosomal RNA [171,172,173,174,175]. *M. tuberculosis* can also resist amikacin through enzymatic modification mediated by the enhanced intracellular survival (Eis) protein, an acetyltransferase enzyme with a unique structure and properties to acetylate aminoglycosides at multiple positions [29,176,177].

Despite the variety of mechanisms of resistance to amikacin detected, the main one found in the clinics is acetylation of the 6′-*N* position. The enzymes that act by this mechanism are called AAC(6′)-I followed by a unique identifier and usually confer resistance to aminoglycosides such as amikacin, tobramycin, and kanamycin but not the gentamicin complex [26,27]. However, exceptions have been detected that show an extended spectrum including gentamicin in their resistance profile [178] or, surprisingly, a reduced susceptibility to quinolones [179]. This family of enzymes includes over 50 representatives that are harbored by Gram-positive or Gram-negative bacteria [26]. These enzymes are also found as fusion proteins located adjacent to the N or C location of the accompanying protein [180], which can be an APH, an ANT, or another AAC [181,182,183,184,185,186,187]. Following we describe representative examples AAC(6′)-I enzymes highlighting some characteristics and their genetic environments. For comprehensive listing and description of AAC(6′)-I enzymes, the reader is referred to previous reviews [26,29,34].

### 3.1. AAC(6′)-I Enzymes of Gram-Positive Bacteria

A small number of 6′-*N*-acetyltransferases with the AAC(6′)-I profile were found in Gram-positive bacteria [184,188,189]. The AAC(6′)-Ie enzyme is fused to the *N*-terminal end of the phosphotransferase APH(2″)-Ia, forming a bifunctional enzyme coded for by the *aac(6′)-Ie-aph(2*″*)-Ia* fusion gene usually located within Tn*4001*-like transposons in Gram-positive bacteria [182,183,190,191,192,193,194]. These transposons have been found in plasmids as well as chromosomes of Gram-positive pathogens such as *S. aureus*, *S. epidermidis*, or *Enterococcus faecalis* [195,196,197,198]. These transposons are characterized by their ability to transpose to random location of Gram-positive chromosomes or plasmids and by the presence of the bifunctional *aac(6′)-Ie-aph(2*″*)-Ia* gene flanked by copies of IS*256* and/or IS*257* in their structure [184]. The crystal structure of the APH(2″)-Ia domain has been determined complexed to GTP analogs, guanosine diphosphate, and aminoglycosides [199,200,201].

The enzyme AAC(6′)-Ii was found in *E. faecium*, its gene is located in the chromosome and confers low levels of resistance, probably as a consequence of the low gene dose [188]. Structural and biochemical characterization of this enzymes permitted to determine that it exists as a homodimer showing subunit cooperativity and the mechanism follows an ordered bi-bi ternary complex with acetyl-CoA binding first [202,203,204].

### 3.2. AAC(6′)-I Enzymes of Gram-Negative Bacteria

In the case of Gram-negative bacteria, the number of AAC(6′)-I enzymes is large, and it is rapidly growing. There were comprehensive reviews that listed the known enzymes at the time they were written [26,27,29]. The latest enzymes of this kind to be reported are listed in Table 1, which continues where the listing in the review by Ramirez and Tolmasky left off [26]. However, in spite of the numerous AAC(6′)-I variants, AAC(6′)-Ib is the enzyme most often found in Gram-negative isolates from the *Acinetobacter* genus, and the *Enterobacteriaceae*, *Pseudomonadaceae*, and *Vibrionaceae* [1]. It should be noted that within these groups of bacteria are those Gram-negatives included in the ESKAPE, the bacteria responsible for the majority of antibiotic resistant hospital infections in the United States [205]. This enzyme is found in numerous variants, most of them differing at the *N*-terminus and some them presenting a few amino acid substitutions that result in enzymes with expanded substrate range. Examples of the later are the AAC(6′)-Ib_11_, which confers resistance to the gentamicin complex, or the AAC(6′)-Ib-cr, which confers a reduced quinolone susceptibility phenotype to the host [178,179,206]. For a detailed description and comparison of amino acid sequences of AAC(6′)-Ib variants, the reader is referred to a recent review [34].

The *aac(6′)-Ib* gene has been found within integrons, transposons, genomic islands, plasmids, and chromosomes [34,39,40,207,208,209,210,211,212,213,214]. It is usually found as a functional, or in some instances deficient, gene cassette that can be located adjacent to the 5′-conserved region or between gene cassettes in the variable region of integrons [215,216,217]. While deficient gene cassettes cannot be mobilized between integrons through the action of the integrase, an alternative mechanism for mobilization of a deficient gene cassette including *aac(6′)-Ib* mediated by homologous recombination was proposed [218].

The earliest reports about *aac(6′)-Ib* identified the gene in plasmids from *S. marcescens* and *K. pneumoniae* [41,42,219,220,221]. In particular, the *K. pneumoniae* plasmid, named pJHCMW1, was exhaustively studied [208,222,223,224]. Its study led to the identification of Tn*1331*, a transposon that harbors four resistance genes, one of them being *aac(6′)-Ib* [40]. This transposon, as well as derivatives, was later found in numerous plasmids from Gram-negatives bacteria. A transposon named Tn*1331.2*, isolated from a *K. pneumoniae* plasmid has a perfect duplication of a 3047-bp DNA segment that includes three resistance genes: the *aac(6′)-Ib*, *ant(3″)-Ia*, and *bla*_OXA-9_ [39]. Tn*6238* is a transposon nearly identical to Tn*1331*, but instead of the *aac(6′)-Ib* gene, it harbors a copy of the *aac(6′)-Ib-cr* variant, product of two point mutations [225]. Tn*1332*, identified in a multidrug-resistant *P. putida* strain, is identical to Tn*1331* with the insertion of three DNA segments, one of which includes a copy of the *bla*_VIM-2_ gene [211]. Another derivative of Tn*1331* with a copy of IS*26* and a deletion that removed part of the *ant(3″)-Ia*, all the *bla*_OXA-9_, and part of the *bla*_TEM-1_ genes was first identified in a 15-kbp plasmid pAAC154 hosted by a carbapenem-resistant ST512 *K. pneumoniae* clinical strain isolated at the Hadassah Hospital, Jerusalem, Israel [226] and then in other plasmids [227,228]. Other derivatives with insertions in Tn*1331* or a deleted version of it added resistance genes to the structure. Insertion of a Tn*4401*-like transposon added a *bla*_KPC_ gene that “upgraded” the mobile element to make it able to confer resistance to carbapenem antibiotics [214,227,228,229,230]. In one instance, a copy of Tn*1331* with an insertion of a Tn*4401*-like and an insertion of Tn*5387*, which includes the fluoroquinolone resistance *qnrB19* gene, was identified in *K. pneumoniae* plasmid [207]. In other cases truncated versions of Tn*1331* were also detected [231,232].

A recent report described the intra- and interspecies transfer of the *aac(6′)-Ib-cr* gene together with *bla*_NDM-1_ by secretion of outer membrane vesicles. A clinical *A. baumannii* strain released vesicles that were purified, and treated with DNase I and proteinase K, before incubation with another *A. baumannii* strain or *E. coli* JM109. Both recipient strains acquired the resistance genes showing that formation and secretion of outer membrane vesicles can be one more natural mechanism of dissemination of resistance genes including *aac(6′)-Ib* [233]. Transfer of plasmids and other cellular components by outer membrane vesicles has been observed before in Gram-negative bacteria [234,235].

### 3.3. Inhibition of Amikacin-Resistance Mediated by AAC(6′)-Ib

Since AAC(6′)-Ib is the major enzyme causing amikacin resistance in Gram-negative pathogens, it is expected that inhibition of its expression or activity would result in reversal of the resistant phenotype. Inhibition of expression of *aac(6′)-Ib* has been researched used antisense technologies, which are inspired by natural mechanisms of control of gene expression and DNA replication [236,237,238,239,240]. Several methodologies use different strategies to interfere with gene expression by supplying a short oligonucleotide or oligonucleotide analog that is complementary to a region of the target gene [241,242,243,244,245,246]. Reduction of levels of resistance to amikacin utilizing antisense oligodeoxynucleotides was first demonstrated targeting single-stranded regions in the mRNA that had been identified by RNase H mapping. Although the mechanisms of inhibition of gene expression remain to be confirmed, all evidence indicates that it occurred by eliciting RNase H-mediated degradation of the RNA moiety of the duplex oligodeoxynucleotide-mRNA [247]. Inhibition of resistance to amikacin in a clinical *A. baumannii* isolate harboring *aac(6′)-Ib* in its chromosome was achieved using an antisense hybrid oligomer consisting of 2′,4′-bridged nucleic acid-NC and deoxyribonucleotide residues conjugated to a permeabilizer peptide that could penetrate the bacterial cells and targeted the initiation of translation region [248]. Another approach that permitted to overcome amikacin resistance was what is known as External Guide Sequence (EGS) technology. It consists of designing antisense molecules that when interacting with the target mRNA acquire a structure that mimics that of a region of a pre-tRNA and elicits digestion by RNase P [244,249]. Gapmers including deoxyribonucleotide residues flanked by locked nucleic acids were potent inducers of RNase P digestion of the mRNA when forming the duplex at the complementary region, and as a consequence, a reduction of levels of antibiotic resistance was observed [250,251,252].

**Figure 3:**
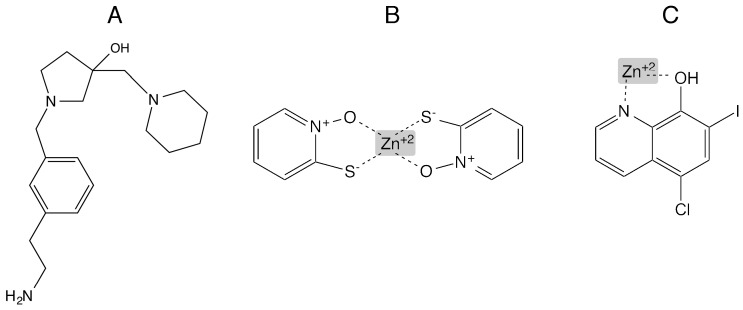
Chemical structures of representative inhibitors of AAC(6′)-Ib-mediated enzymatic acetylation of amikacin. (**A**) 1-[3-(2-aminoethyl)benzyl]-3-(piperidin-1-ylmethyl)pyrrolidin-3-ol; (**B**) zinc pyrithione coordination complex; (**C**) zinc clioquinol coordination complex [267].

Numerous kinds of compounds with robust inhibitory activity of AAC(6′)-I enzymes have been described and are listed in various comprehensive reviews [26,49,54,253]. An inhibitor of AAC(6′)-Ib was first designed by an NMR-fragment based-approach [254]. Later, inhibitors of the same enzyme were identified using glide [255,256] and Autodock Vina 1.1.2 [257] computer docking programs. Compounds of different chemical nature but that behave as robust inhibitors of the enzymatic acetylation catalyzed by AAC(6′)-Ib. However, only one identified with the glide software, 1-[3-(2-aminoethyl)benzyl]-3-(piperidin-1-ylmethyl)pyrrolidin-3-ol (Figure 3) showed inhibitory activity of resistance to amikacin in cells growing in culture [258,259]. An acetyltransferase responsible for resistance to amikacin and other aminoglycosides present in resistant *M. tuberculosis* isolates attracted considerable interest in finding inhibitors of the enzymatic inactivation. These efforts resulted in isolation of various inhibitors that reduced the levels of amikacin resistance in growing cells [260,261,262,263]. Another group of compounds that were found to inhibit the acetylation reaction is integrated by Zn^+2^ and other metal ions [264,265,266]. Although the mechanism of this inhibition is still unknown, an attractive hypothesis is that it occurs through formation of a coordination complex between the substrate aminoglycoside and the cation that is no longer a suitable substrate of the enzyme. The concentrations of metal ions, Zn^+2^ or Cu^+2^, necessary for reversing resistance to amikacin in clinical and laboratory *A. baumannii*, *K. pneumoniae*, and *E. coli* strains in liquid cultures are in the low mM levels. However, when the metals are added in complex with some ionophores such as pyrithione or clioquinol, a small hydrophobic molecule also being investigated as a candidate drug to treat tumors and neurodegenerative diseases [267,268], low μM levels are sufficient for phenotypic conversion to susceptibility to amikacin [264,266,269].

## 4. Final Remarks

Aminoglycosides are one of the first kinds of antibiotics discovered dating back to the 1940s. They are an essential component of the armamentarium against serious infections caused by Gram-negative as well as Gram-positive bacteria; in this latter case, they are usually administered in combination with other antibiotics. Although the first representatives of this family were of natural origin, further research stimulated by the emergence of aminoglycoside modifying enzymes that confer resistance and disseminate very quickly, resulted in the design of a generation of semisynthetic members that are refractory to enzymatic inactivation. Amikacin, introduced in the late 1970s, was and continues to be an essential antibiotic used against numerous infections caused by multidrug-resistant organisms. Unfortunately, the AAC(6′)-I enzymes, and in particular the AAC(6′)-Ib, threaten to reduce the efficacy of amikacin. However, research efforts to design new semisynthetic molecules such as plazomicin or inhibitors of the expression or action of AAC(6′)-Ib, give hope that we will continue to count on aminoglycosides to fight severe multiresistant infections.

## Figures and Tables

**Table 1:** Newer AAC(6′)-I proteins ^1^.

Enzyme	Genetic Location	Accession Number	Host	Reference
AAC(6′)-Iag	Integron (In124)-Plasmid	AB472901	*P. aeruginosa*	[270]
AAC(6′)-Iai	Integron	EU886977	*P. aeruginosa*	[271]
AAC(6′)-Iaj	Integron (In151)-Chromosome	AB709942	*P. aeruginosa*	[272]
AAC(6′)-Iak	Chromosome	AB894482	*Stenotrophomonas maltophilia*	[273]
AAC(6′)-Ial	Chromosome	AB894481	*S. marcescens*	[274]
AAC(6′)-Ian	Plasmid	AP014611	*S. marcescens*	[275]
AAC(6′)-Iap ^2^		AB979699	*S. marcescens*	[274]

^1^ A complete listing can be found in [26]; ^2^ The protein named AAC(6′)-Iap in [274] is named different in GenBank under the stated accession number.
